# Identification of novel molecular drivers, prognostic and diagnostic biomarkers for Inflammatory Bowel Disease (IBD): protocol for the Nottingham/AstraZeneca prospective IBD observational cohort study

**DOI:** 10.1136/bmjopen-2025-105790

**Published:** 2025-11-23

**Authors:** Ana Lilia Serna-Valverde, Eva Rodriguez-Suarez, Daniel J B Marks, Ulf Gehrmann, Jessica Neisen, Sarah Clarke, Thean Soon Chew, Fraser Cummings, Shanika De Silva, John Nicholas Gordon, Paul Knight, Jimmy Limdi, Kamal Patel, Benjamin Crooks, Shaji Sebastian, Christos Polytarchou, Nicholas R F Hannan, Gordon W Moran

**Affiliations:** 1 Translational Medical Sciences, University of Nottingham School of Medicine, Nottingham, UK; 2 Translational Sciences and Experimental Medicine, AstraZeneca UK Limited, Cambridge, UK; 3 Early Clinical Development (Immunology), AstraZeneca UK Limited, Cambridge, UK; 4 Translational Sciences and Experimental Medicine, AstraZeneca UK Limited, Gothenburg, Sweden; 5 Sheffield Teaching Hospitals NHS Foundation Trust, Sheffield, UK; 6 Academic Unit of Gastroenterology, The University of Sheffield, Sheffield, UK; 7 Gastroenterology, Southampton University Hospitals NHS Trust, Southampton, UK; 8 The Dudley Group NHS Foundation Trust, Dudley, UK; 9 Hampshire Hospitals NHS Foundation Trust, Winchester, UK; 10 Manchester University NHS Foundation Trust, Manchester, UK; 11 Gastroenterology, Northern Care Alliance NHS Foundation Trust, Manchester, UK; 12 Department of Gastroenterology, St. George’s University Hospitals NHS Foundation Trust, London, UK; 13 Bolton NHS Foundation Trust, Bolton, UK; 14 Hull University Teaching Hospitals NHS Trust, Hull, UK; 15 Department of Biosciences, Centre for Health, Ageing and Understanding Disease (CHAUD), Nottingham Trent University School of Science and Technology, Nottingham, UK; 16 Nottingham University Hospitals NHS Trust, Nottingham, UK

**Keywords:** Inflammatory bowel disease, MOLECULAR BIOLOGY, Treatment Outcome

## Abstract

**Abstract:**

**Introduction:**

Crohn’s disease (CD) and ulcerative colitis (UC) are chronic, inflammatory bowel diseases (IBDs) of unknown origin, affecting the gastrointestinal tract and often causing extraintestinal symptoms. Conventional treatments (eg, glucocorticosteroids, immunomodulators) and targeted advanced treatments, including anti-TNFα, antibodies to p40 subunit of IL-12/23, antibodies to p19 subunit of IL-23, anti-α4β7 integrin, Janus kinase inhibitors (JAKis) and sphingosine-1-phosphate receptor (S1PR) modulators, do not achieve sustained responses for all patients, leaving significant unmet therapeutic needs.

**Methods and analysis:**

This prospective, multi-centre observational study will follow a cohort of 240 patients across multiple study centres within NHS trusts in the UK who are initiating or switching biologics, specifically anti-TNFα and anti-α4β7 integrin for UC, and anti-TNFα, antibodies to p40 subunit of IL-12/2 and JAKi for CD. Through comprehensive profiling of immunological, transcriptional, microbiome, genetic and proteomic markers at baseline, week 12, and week 52, this study aims to uncover non-invasive biomarkers that predict response to these drug classes, ultimately advancing personalised medicine in IBD.

**Ethics and dissemination:**

Ethical approval for the Nottingham/AstraZeneca study was granted by the West of Scotland Research Ethics Committee. Recruitment began in December 2022 and is currently ongoing at 10 NHS Trust sites across the UK. Study findings will be disseminated by publication in peer-reviewed journals and presentations at relevant national and international conferences.

**STRENGTHS AND LIMITATIONS OF THIS STUDY**

- Objective assessment of disease activity on patient inclusion through ileocolonoscopy.
- Deep phenotyping through RNA sequencing, proteomics and peripheral blood mononuclear cells (PBMCs) extraction.
- Longitudinal follow-up to 12 months.
- Class of antibodies to the p19 subunit of Il-23 not represented.
- Due to the requirement of endoscopy, participant numbers are limited to 240.

## Introduction

 Dysregulation of immune responses can lead to an imbalance between pro-inflammatory and anti-inflammatory signals, exacerbating tissue damage and symptoms.^1^ Circulating immune cells and resident memory T cells play crucial roles in the pathogenesis of inflammatory bowel disease (IBD).^2^,^4^ These cells, including T cells, B cells, neutrophils and monocytes, become activated and migrate to the inflamed gastrointestinal tract, contributing to chronic inflammation.^5^ Furthermore, specific immune cell subsets, such as Th17/Treg cells, have been linked to disease severity and response to therapy.^6^ Similarly, the interplay between immune cells and epithelial tissue is critical for disease progression. Immune cells, such as T cells and monocytes, infiltrate the intestinal epithelium, triggering inflammatory responses that damage epithelial cells and further disrupt a potentially dysfunctional gut barrier.^7 8^ Understanding the profile and characteristics of tissue-resident and circulating immune cells and how they interact with epithelial and submucosal environment in IBD is vital for uncovering disease mechanisms and developing new therapies for patients in whom existing therapies still do not deliver high levels of remission.

Present-day decisions on treatment options for patients with Crohn’s disease (CD) and ulcerative colitis (UC) are usually guided by disease activity and severity, location of bowel inflammation and the presence of other clinical features (such as extraintestinal manifestations, fistulae and malabsorption). The therapeutic options currently available include ‘conventional treatments’ including glucocorticosteroids, 5-aminosalicylates and immunomodulators (azathioprine, 6-mercaptopurine and methotrexate). Over recent years, targeted biologic treatments, such as TNFα antagonists, integrin antagonists, antibodies to p40 subunit of IL-12/23, antibodies to p19 subunit of IL-2, Janus Kinase inhibitors (JAKis) and sphingosine-1-phosphate receptor (S1PR) modulators, have become available to block the maladaptive immune response believed to be central to IBD pathogenesis.^9 10^

Despite the availability of these treatments, not all patients with IBD will respond or maintain their response. For example, approximately 40% of patients do not respond initially to TNFα antagonists, and up to 46% of responding patients lose response over time.^11^ Lack of response to agents in other classes is similar and tends to decline further with each additional line of therapy.^12^ A chronic and unbalanced inflammatory response leads to disease complications in a substantial number of patients that has a negative impact on quality of life and an increased need for surgical interventions. A third of patients with CD eventually require a surgical intervention within the first decade after diagnosis, and approximately 15% of UC patients may require a colectomy.^13^

With annual direct costs of severe IBD relapses related to hospitalisation being £10 513 for CD and £10 760 for UC on average per patient, there is an urgent need for treatment algorithms to enable precise and personalised treatment.^14^

The development of optimal treatment strategies to stratify patients based on their molecular endotypes, enabling the selection of the most effective therapy for each subgroup, has been identified as a top research priority for IBD by the UK priority-setting partnership with the James Lind Alliance.^15^

This study will follow the treatment response journey as patients switch onto or between advanced therapies as part of standard clinical practice. For feasibility reasons, we will initially target patients who are initiating TNFs and vedolizumab in UC and TNFs, ustekinumab and upadacitinib in CD. By profiling immunological, transcriptional, microbiome, genetic and proteomic biomarkers at baseline and following advanced treatment initiation or switch, this study aims to identify prognostic and/or diagnostic biomarkers for CD and UC in order to inform the development of new therapeutic strategies for IBD.

## Methods and analysis

### Study objectives

The primary study objective is to perform bulk and single-cell RNA sequencing on both peripheral blood mononuclear cells (PBMCs) isolated from whole blood and intestinal epithelial cells from inflamed and non-inflamed gut tissue biopsies of the same patient. The patients will provide samples before and after starting advanced treatments. Analysis of transcriptomic profiles will allow us to (1) Dissect the cellular and molecular mechanisms associated with the intestinal inflammation of each patient, (2) Understand cell-cell interactions at the level of the intestinal epithelium, (3) Identify correlates of these mechanisms in the composition and molecular profile of the peripheral blood cell subsets and (4) Elucidate the effect of the assigned treatment on PBMC composition and cellular transcriptional phenotypes. The findings will be analysed and stratified by drug-responder status to better understand which biological pathways are associated with each subtype of inflammation.

The secondary objective is to phenotype PBMC from participants at enrolment and at the 3-month follow-up visit in order to gain a better understanding of how the immune cell compartments are affected by (a) Diagnosis, (b) Disease activity, (c) Ongoing and previous treatment, (d) Concomitant diseases and (e) Demographic variables and other clinical data.

A tertiary objective will be the identification of non-invasive biomarkers that reflect the underlying molecular mechanisms of disease and may serve as potential future prognostic/diagnostic biomarkers.

### Study design

**Figure 1:**
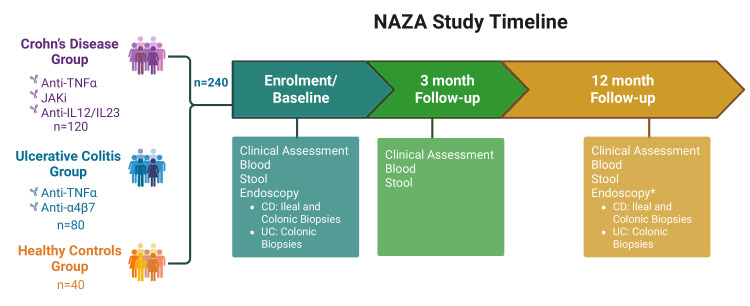
Nottingham/AstraZeneca study design schematic. Participants with active inflammatory bowel disease (IBD) who are initiating or switching to a new targeted biologic therapy will be recruited for the study. The cohort of 240 participants will consist of 120 patients with Crohn’s disease, 80 patients with ulcerative colitis and 40 non-IBD participants. Enrolment and baseline visits may occur during a single visit for convenience or can be conducted separately, provided that the enrolment visit takes place within 1 month prior to the baseline visit. Follow-up appointments at 3 months and 12 months will be scheduled from the start of the new biologic therapy rather than from the baseline appointment. A thorough clinical assessment and biomarker sampling will be conducted at the baseline, 3-month and 12-month visits. Endoscopic data will be collected at baseline, and the 12-month endoscopy will be optional, as clinically indicated. Anti-TNFα, tumour necrosis factor alpha antagonists (Infliximab or Adalimumab); Anti-IL-12/23, interleukin 12 and 23 antagonist (Ustekinumab); Anti- α4β7, integrin α4β7 antagonist (Vedolizumab); CD, Crohn’s Disease; JAKi, Janus Kinase inhibitor (Upadacitinib); UC, Ulcerative Colitis.

The Nottingham/AstraZeneca (NAZA) study is a multicentre prospective cohort study led by the University of Nottingham and funded by AstraZeneca; its design is illustrated in figure 1. The study will recruit participants with moderate to severely active CD or UC (see box 1 for eligibility criteria) who are either starting or switching to a new targeted advanced therapy and are undergoing routine endoscopic assessments as part of standard clinical care. Eligible advanced therapies for these participants include anti-TNFα therapy, anti-α4β7 (vedolizumab) for UC, and either JAKi (upadacitinib) or anti-IL-12/23 (ustekinumab) for CD. Vedolizumab was initially included for CD but was replaced early in the study with the JAKi, upadacitinib, for feasibility reasons. Participants who are switching therapies must be changing to a treatment with a different mechanism of action. Additionally, the study will include a control group of non-IBD participants who are scheduled for a colonoscopy as part of standard care and have no pathology found during the examination. Comprehensive clinical data will be collected along with stool and blood samples at three key points: before starting treatment (baseline), and during follow-up visits at month 3 and month 12 after initiating treatment. Additionally, endoscopic data and gut tissue biopsies will be collected at baseline and, if clinically indicated, again 12 months after the start of the biologic therapy. If a patient fails to respond to the new biologic and the clinical team decides to discontinue the treatment, the 12-month follow-up appointment will be brought forward and completed as an Early Termination Visit.

Box 1 **Eligibility criteria for Nottingham/AstraZeneca study**

**Nottingham/AstraZeneca study eligibility criteria**

**Inclusion criteria**

*General requirements:*

- Provision of signed and dated, written informed consent before any study-specific procedures.
- Participants must be aged 16 years or older.
- Inflammatory bowel disease (IBD) patients must be booked for colonoscopy or flexible sigmoidoscopy as part of their standard care.
- IBD patients must be starting or switching to a new mechanism of action which includes:
  - For Crohn’s Disease (CD): anti-TNFα therapy, Upadacitinib or Ustekinumab.
  - For Ulcerative Colitis (UC): anti-TNFα therapy or Vedolizumab.

*Crohn’s disease group:*

- Active CD defined by one of the following:
  - Inflammatory markers:
    - C-reactive protein (CRP) ≥5 mg/L, or
    - Faecal calprotectin (FCP) ≥250 μg/g, or
  - Endoscopy findings:
    - Visible ulcerations on ileocolonoscopy with a total Simple Endoscopic Score for Crohn’s Disease ≥7, or ≥4 if disease is confined to the terminal ileum, or
  - Imaging:
    - Visible active disease on cross-sectional imaging.

*Ulcerative colitis group:*

- Active UC defined by one of the following:
  - Inflammatory markers:
    - CRP ≥5 mg/L, or
    - FCP ≥250 μg/g, or
  - Endoscopy findings:
    - Mayo endoscopy sub-score ≥2.

*Healthy controls group:*

Participants without IBD who are attending a lower gastrointestinal colonoscopy, where no pathology is found on examination.

**Exclusion criteria**

*All groups:*

- Inability to provide informed consent.
- Positive screening results for hepatitis B surface antigen, hepatitis C or HIV.
- Ongoing infection requiring treatment.
- Active COVID-19 infection, as determined by local standard care procedures.
- Participation in another clinical study with pharmacological intervention within 3 months prior to the baseline visit.
- Current diagnosis of cancer.
- Previous solid organ or stem cell transplant.
- Recent transfusion of blood, plasma or platelets within 120 days prior to enrolment.
- Pregnancy.
- Investigator’s clinical judgement that the patient should not participate in the study.
- For healthy controls, a prior history of any form of IBD (CD, UC, microscopic colitis, indeterminate colitis or IBD-unspecified) applies. Additionally, all general exclusion criteria apply.

**Figure 2:**
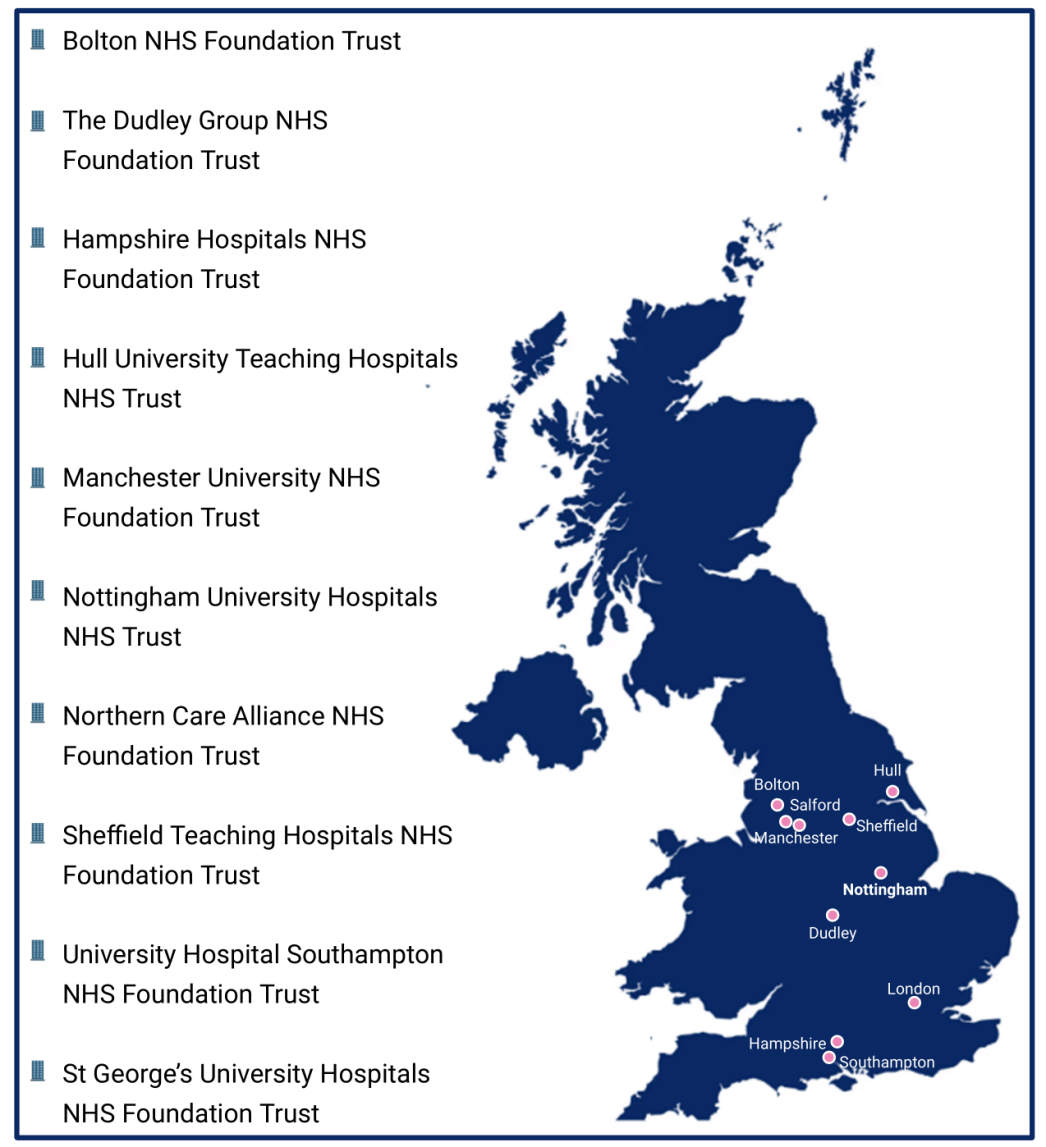
UK NHS trust sites recruiting for the Nottingham/AstraZeneca (NAZA) study. The map illustrates the 10 NHS trust sites across the UK that are actively recruiting for the NAZA study. These include Bolton NHS Foundation Trust, The Dudley Group NHS Foundation Trust, Hampshire Hospitals NHS Foundation Trust, Hull University Teaching Hospitals NHS Trust, Manchester University NHS Foundation Trust, Nottingham University Hospitals NHS Trust, Northern Care Alliance NHS Foundation Trust, Sheffield Teaching Hospitals NHS Foundation Trust, University Hospital Southampton NHS Foundation Trust and St. George’s University Hospitals NHS Foundation Trust.

This study aims to recruit a total of 240 participants, including 120 patients with CD, 80 patients with UC and 40 healthy individuals. Recruitment will occur at 10 study centres within NHS trusts across the UK (figure 2). The University of Nottingham study centre will lead recruitment, oversee data analysis and manage sample storage. Additional recruitment and initial sample storage will be handled by the collaborating NHS Trusts, ensuring proper sample storage until transfer to the University of Nottingham. Serum samples will then be sent to AstraZeneca laboratories for proteomic analysis, while stool samples will be sent to Nottingham Trent University for microbial analysis. All remaining samples and associated data will be securely stored at the University of Nottingham. The study will span 42 months and will conclude 12 months after the last participant is recruited. This timeframe is designed to ensure the thorough completion of laboratory analyses following the final visit of the last participant. Recruitment began in December 2022 and is currently ongoing, with the predicted end of recruitment in July 2026 and the completion of follow-up in July 2027. Once these analyses are finished, any samples for which participants have provided consent for future research will be transferred to the University of Nottingham research tissue bank, where they will be available for use in future IBD research.

### Study management

Overall responsibility for the study will be held by the Chief Investigator, who will oversee all aspects of study management and serve as the data custodian. Monitoring of the study will be conducted by a Trial Management Group (TMG), which will meet at least once a month, or more frequently as necessary. The Nottingham Biomedical Research Centre (BRC) will serve as the central coordinating centre for the study, arranging trial monitoring, audits and all TMG meetings. Together, the TMG and BRC will ensure that the trial is conducted under Good Clinical Practice (GCP) principles and relevant regulations. The TMG will closely monitor data accumulation, safety and ethical issues, and verify compliance with the study protocol. Furthermore, the BRC will ensure secure storage of all essential study documents, including the Investigator Site File, until the completion of the study. Afterwards, these documents will be retained for a minimum of 15 years.

### Selection of study participants

A collaborative network comprised of the chief investigator, clinical consultants and nurses with expertise in IBD will work together to recruit IBD patients who attend gastroenterology clinics in Nottingham University Hospitals NHS Trust and other NHS Trust sites. The planned recruitment for the NAZA study aims to enrol a total of 240 participants. To achieve an even distribution across the study groups, approximately 40 participants, but no fewer than 30, should be recruited into each treatment arm. This includes 40 non-IBD participants, 40 participants with UC receiving anti-TNFα therapy and 40 receiving vedolizumab. For CD, the study aims to recruit 40 participants treated with anti-TNFα therapy, 40 with upadacitinib and 40 with ustekinumab. Additionally, CD participants will be stratified by disease location, with a target minimum of 20% from each location: ileal, colonic or ileocolonic. In total, this recruitment plan will comprise 40 healthy participants, 80 participants with UC and 120 participants with CD.

Recruitment rates will be continuously monitored, and the target number of patients per study group will be revised across the collaborating centres to ensure the timely completion of the study. The recruited non-IBD participants will be involved for a maximum of 4 weeks, while participants with CD or UC will engage in the study for approximately 12 months, with samples collected at baseline and again at three and twelve months after initiating the new biologic therapy. If the clinical team determines that a participant is not responding to the new biologic treatment before the 12-month follow-up visit, treatment will be discontinued. In this case, the follow-up visit will be rescheduled to serve as the early termination visit (ET), and it will be conducted as soon as feasible for the patient. If participants are withdrawn from the study after enrolment, they may be replaced with new participants at the discretion of the study sponsor and principal investigator, with each replacement assigned a new enrolment number. Where appropriate, consent will be obtained to include retained data from withdrawn participants in the final analyses, and participants may request the destruction of any remaining tissue samples.

### Eligibility criteria

Patients who, in the investigator’s clinical opinion, have moderate to severely active IBD, are switching to a new biologic therapy and are scheduled for routine endoscopic assessments will be approached by a member of their clinical care team to assess their eligibility for the study. Individuals will qualify for enrolment if they fulfil all inclusion criteria and do not meet any of the exclusion criteria listed on box 1.

### Sample size and justification

The NAZA study aims to identify multi-omic biological drivers of disease progression and treatment response in IBD. Given its exploratory nature, estimating a statistically powered sample size is challenging. However, a cohort of 240 participants is considered to be adequate for meaningful insights into transcriptional, proteomic and microbiome alterations associated with the disease. Similar participant numbers have earlier been able to identify transcriptional signatures of response in IBD. For instance, in an earlier unpublished study by our group, preliminary data from in-house immunohistochemistry analysis has identified a mean difference of ~110 CCR9^+^ cells/mm^2^ in inflamed and non-inflamed gut tissue from CD patients. Based on these data and the minimum number of available patients per disease severity category, a sample size of at least 34 inflamed versus non-inflamed biopsy pairs from CD patients provided >99% power to detect a difference in the mean number of CCR9^+^ cells in the gut tissue of 110 cells/mm^2^ between severe disease patients and patients in remission assuming a SD of 70 cells/mm^2^ with a two-sided 5% level of statistical significance. The sample size also provided 90% power to detect a difference in the mean number of CCR9^+^ cells between mild and severe disease patients of 60 cells/mm^2^. Thus, a sample size of 40 patients per treatment arm is considered sufficient to detect biologically meaningful biomarker differences between samples collected for the NAZA study.

### Study procedures and regimen

Potential IBD study participants will be identified through pre-screening at IBD clinics or during routine clinical appointments, while healthy controls will be selected from individuals without IBD who are scheduled for colonoscopy at hospitals or clinics. All eligible individuals will receive a patient information sheet and an invitation letter detailing study participation. A member of their clinical team will then reach out to explain the details of the trial and assess their interest. Patients will be informed that trial enrolment is voluntary and will not impact their treatment or care. They may withdraw from the study at any time, with previously collected data and samples retained unless they request their removal. To ensure that individuals identified through either pathway can make an informed decision, they will have as much time as needed to review the provided information and ask any questions. For those who decide to participate, an enrolment appointment will be scheduled, ideally coinciding with a routine clinic visit where feasible.

Once eligibility is confirmed, a trained team member will obtain informed consent from each participant prior to trial entry and before any study-related interventions, including physical examination and clinical history taking. The informed consent form will be signed and dated by the participant, with copies retained by the participant, the investigator and the patient’s hospital records. If any subsequent amendments are made to the final protocol that could affect a participant’s involvement in the trial, updated consent will be obtained through an amended consent form, which the participant will be required to sign.

**Figure 3:**
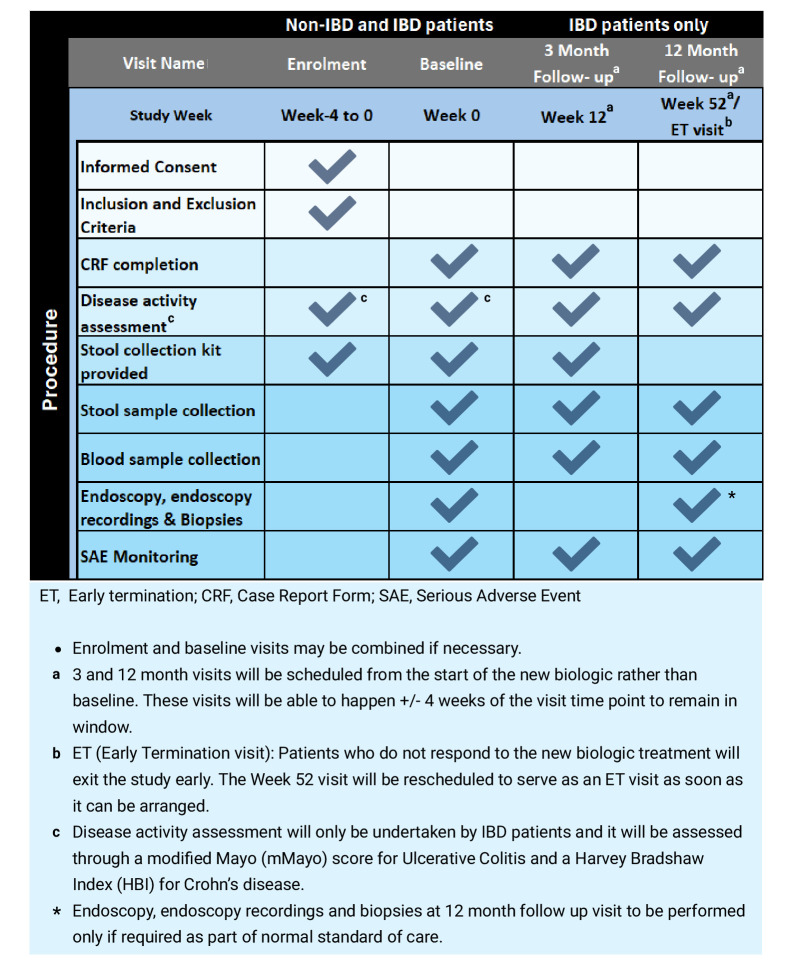
Schedule of procedures for Nottingham/AstraZeneca (NAZA) study. Outline of study procedures for non-IBD and IBD participants in the NAZA study, spanning from enrolment to the 12-month follow-up. These procedures will be conducted by staff at Nottingham University Hospitals NHS Trust and collaborating co-investigator sites. CRF, Case Report Form.

Recruited participants will complete data collection following the provision of informed consent and before starting treatment during the baseline visit, as well as at week 12 and 52 after commencing biologic therapy, as shown in figure 3. Data collected at these three time points will include the completion of Case Report Forms (CRFs), which will capture comprehensive clinical information and IBD symptom scores, along with biomarker sampling of blood and stool. Disease activity will be assessed through a modified Mayo (mMayo) score for UC and a Harvey Bradshaw index for CD. These assessments will only be undertaken by IBD patients. Endoscopic biomarker sampling will be conducted at baseline and, if endoscopy is performed as part of standard care, again 12 months after the initiation of biologic therapy. All research sampling will adhere to the standard care protocols established by NHS guidelines. Additionally, all medications relevant to IBD treatment, including both prescription and over-the-counter drugs, will be documented in the CRF at each visit, noting any prior biologic treatment failures due to primary or secondary non-response or drug intolerability.

### Study variables

Sample collection and handling will follow standardised protocols, with full requirements specified in the NAZA study laboratory manual. The investigator is responsible for ensuring that all data are collected and documented in a timely manner, in adherence to provided guidelines, and will keep records of individuals considered for enrolment but not enrolled, to verify unbiased participant selection. Informed consent must be obtained from all participants prior to any study-specific procedures. For those participants meeting the eligibility criteria and scheduled for a disease assessment endoscopy before therapy, consent must be secured in advance to allow for the collection of research biopsies during the procedure.

#### Screening and demographic measurements

At enrolment, the following data will be captured in the CRF: informed consent, demographic information (year of birth, sex and ethnicity), height, weight and nutritional habits (eg, vegetarian, vegan). A standard medical history will record comorbidities, past diseases, surgeries and smoking status. Additionally, infection history for Hepatitis B, Hepatitis C, HIV and SARS-CoV-2 will be documented. Information on prior and current medications will be updated at each follow-up visit, along with symptom scores and endoscopy results, if applicable.

Endoscopy assessments in the NAZA study will be conducted at baseline and, if clinically indicated, at the 12-month follow-up, using the SES-CD scoring system for CD and the mMayo scoring system for UC. For both conditions, endoscopy results may be derived from video recordings of the procedures.

#### Biomarkers

**Figure 4:**
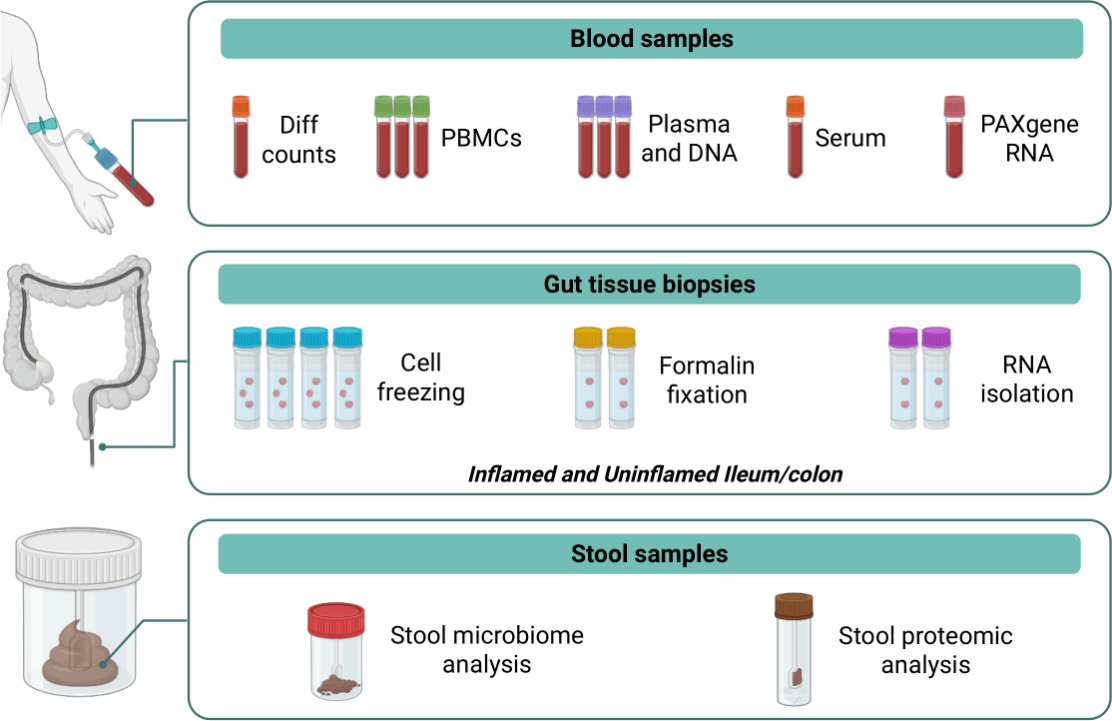
Collection of biomarkers for Nottingham/AstraZeneca study. Gut tissue samples collected in this study will undergo formalin fixation, cryopreservation or processing for RNA isolation. Matched blood and stool samples will be collected from all participants. Biopsy samples will be obtained during the baseline endoscopy and at the 12-month follow-up, only if endoscopy is required as part of standard care. Blood samples will be collected at baseline, 3-month and 12-month follow-ups for use in differential cell counts, PBMC extraction, plasma extraction, serum analysis and DNA and RNA analysis. Participants will also provide two stool sample tubes, collected using provided kits, for microbiome and proteomic analyses at baseline, 3-month and 12-month follow-ups.

Biological samples collected in this study include gut tissue biopsies, blood and stool samples, as illustrated in figure 4. The CD and UC cohorts will undergo phenotyping at both baseline and follow-up visits, while the non-IBD cohort will be phenotyped only at baseline, in accordance with the IBD group protocol.

Biopsy samples will be collected from the proximal-distal gradient, including inflamed and non-inflamed tissues when applicable. The total number of biopsies will vary based on the participant’s cohort (CD/UC/non-IBD), with a maximum of 10 pinch biopsies per site. For CD patients, up to 40 biopsies may be taken from four sites, including 10 from inflamed ileum, 10 from non-inflamed ileum, 10 from inflamed colorectal segments and 10 from non-inflamed colorectal segments. For UC patients, up to 20 biopsies will be taken from two sites, with 10 from inflamed colorectal segments and 10 from non-inflamed colorectal segments. In cases of pancolitis, 10 biopsies will be taken from the inflamed colon. For non-IBD participants, up to 10 biopsies from the ileum and 10 from colorectal segments will be collected for research purposes; however, if any significant pathology or abnormality is detected during colonoscopy, no research samples will be collected, and the participant will be withdrawn from the study. The collected gut tissue samples in this study will be formalin-fixed for paraffin embedding, cryopreserved or processed for bulk RNA sequencing.

Matched blood and stool samples will be collected at baseline, week 12 and week 52 visits. Approximately 60 mL of blood will be drawn by a qualified practitioner to be used for differential cell counts, PBMC extraction, plasma extraction, serum analysis and DNA and RNA analysis. Differential cell counts will be performed locally at each NHS Trust site, with results accepted from the previous 12 weeks. Alongside blood samples, participants will also collect stool samples at each assessment using provided kits: one for microbiome analysis (OMNIgene-GUT tube) and one for proteomic analysis (universal polystyrene tube). Stool samples should be collected at home before bowel preparation for endoscopy. If enrolled during endoscopy, stool samples must be taken at least 48 hours after the procedure and prior to starting any new therapy; however, this collection should not delay therapy initiation.

All collected samples will be labelled at the investigator site with a unique identifier, ensuring traceability throughout the sample’s lifecycle until disposal. Samples will be transported and stored as specified in the laboratory manual. After analysis, any remaining samples or derivatives will be stored at the University of Nottingham study centre for use in future research.

### Transport and storage of the tissues

Samples collected in this study, including gut tissue biopsies, blood and stool, will be anonymised and labelled with a unique identifier linked to study data and consent form. These biosamples will be transported from the collection point following University Biosafety Guidelines and stored at the Nottingham Digestive Diseases Centre laboratories in a Human Tissue Authority licensed facility (No. 12265) before analysis. Each sample will then be processed according to protocols in the laboratory manual, with analyses conducted at AstraZeneca R&D sites (Cambridge, UK; Gothenburg, Sweden; Gaithersburg, Maryland, USA), the University of Nottingham, or other collaborating facilities.

**Figure 5:**
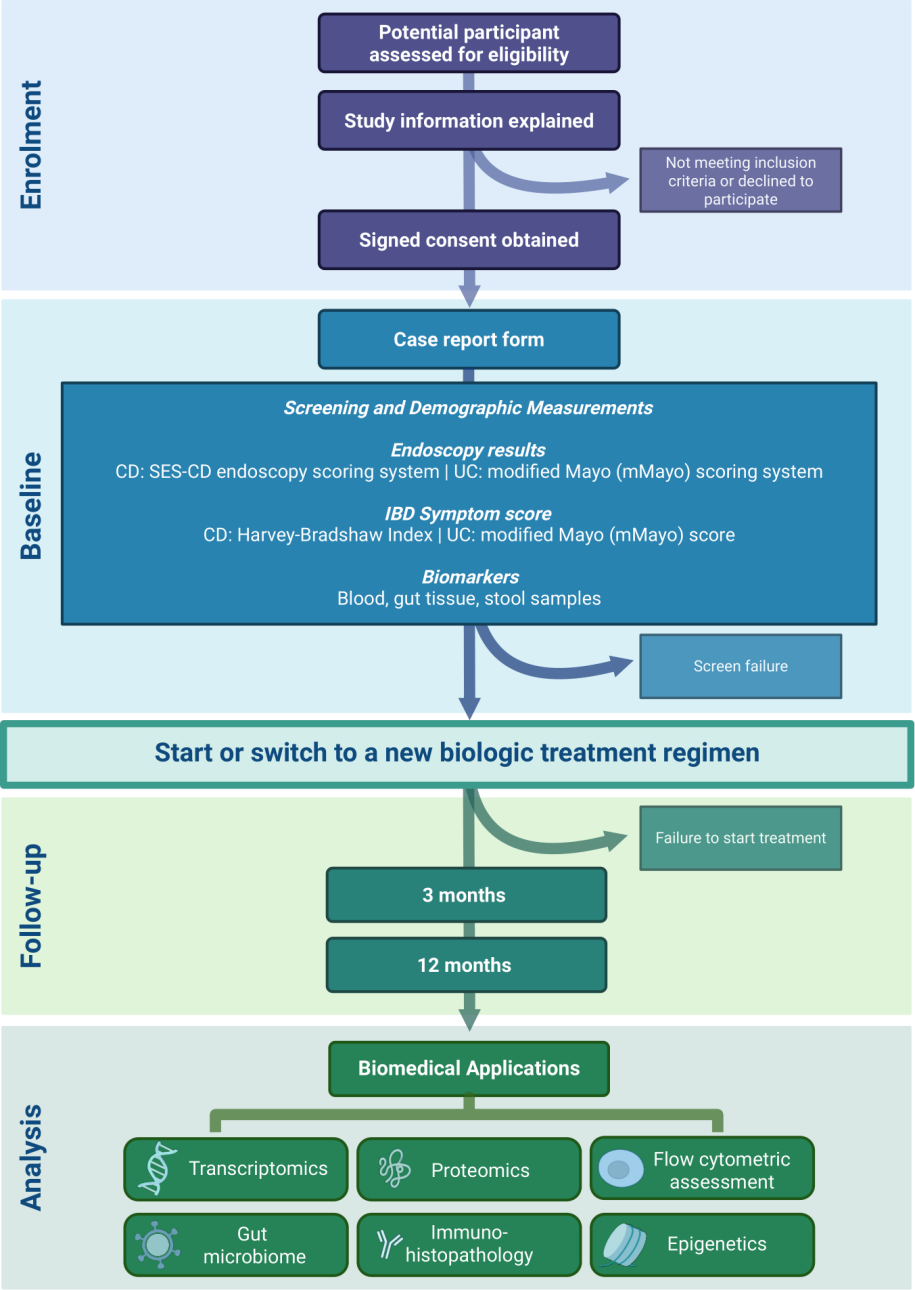
Nottingham/AstraZeneca study overview. Process flow diagram illustrating an overview of study activities, including enrolment, baseline assessments, follow-up visits and data analysis for biomedical applications. CD, Crohn’s disease; IBD, inflammatory bowel disease; SES-CD, Simple Endoscopic Score for Crohn’s Disease; UC, ulcerative colitis.

A full chain of custody will be maintained for all samples throughout their lifecycle. Investigators and site personnel will maintain complete traceability of samples from collection through storage and shipment, documenting all receipts. On receipt at the analysis site or laboratory, the receiving facility will log the arrival, maintain traceability through use and storage, and keep detailed records of each sample’s status until it is used, disposed of, or shipped further if necessary. Samples will be kept in −80°C freezers, under yearly service contract and monitoring system to ensure their integrity. Specific procedures will be followed depending on sample type: gut tissue samples will be processed at the University of Nottingham’s Biodiscovery Institute, stool samples will be transferred to Nottingham Trent University for proteomic analysis, and plasma/serum samples will be sent to AstraZeneca for further processing and proteomic studies. All relevant data generated from the study will be stored at the University of Nottingham. An overview of the NAZA project, detailing the process from patient recruitment to sample processing and analysis, is illustrated in figure 5.

On study completion, samples will either be stored for future research or destroyed depending on participant consent. Samples with participant approval will be stored in the Research Tissue Bank (Licence No. 12265) for potential future research, with some shared with AstraZeneca. For participants who decline future use, samples will be disposed of according to the Human Tissue Act, 2004. Findings from future analyses will not be part of the formal Study Report but may be published in peer-reviewed journals or presented at scientific congresses.

If a participant withdraws consent for the use of their donated biological samples, those samples will be disposed of or destroyed if they have not already been analysed and documented. Since the collection of these samples is integral to the study, the participant will be withdrawn from further participation. The Principal Investigator will immediately notify the sponsor of the withdrawal and ensure that any biological samples stored at the study site are identified, disposed of and these actions are documented. Additionally, laboratories holding the samples will be informed of the withdrawn consent, and they too will dispose of the samples and document the action. The sponsor will ensure that any central laboratories holding samples are instructed to dispose and that the samples are disposed of accordingly. If the samples have already been analysed, AstraZeneca and the University of Nottingham are not required to destroy the resulting data. Data collected up to the point of withdrawal may still be used in the study, and consent will be sought to include this data in final analyses. Participants may also request the destruction of any remaining tissue samples.

### Statistical analysis

As this study does not include an investigational medicinal product and is not designed for regulatory submission (although it may be used in support of such a submission), the methods of statistical analyses will be determined depending on the nature of the data collected.

### Potential future benefit to patients

We envision that the findings from the NAZA study will uncover new molecular targets and prognostic or diagnostic biomarkers for CD and UC, ultimately aiding in the development of innovative therapeutic strategies for IBD.

In addition to these advancements, specific projects stemming from the NAZA study hold great promise for enhancing patient care. The first project involves the development and curation of systematically annotated endoscopy video frames, linked to treatment response. This will create a robust dataset to explore novel endoscopy scoring tools to enable more accurate monitoring of disease activity and explore prediction of therapeutic efficacy, allowing for timely interventions.

A second project focuses on creating an advanced organoid model for IBD that will run parallel to the NAZA cohort. This engineered in vitro platform will mimic the inflammatory processes associated with IBD, facilitating the investigation of molecular alignment with both the data exchange platform, IBD Plexus dataset and NAZA patient data.^16^ By studying the organoid model’s responses to compounds used in the NAZA study, we aim to correlate these responses with emerging clinical results. Furthermore, the data gathered from these experiments may inform the development of Quantitative Systems Pharmacology models, enhancing our understanding of drug interactions and treatment outcomes. Together, these initiatives could significantly improve personalised treatment approaches and patient outcomes in IBD.

A third project will involve multiplex imaging to enhance 2-dimensional understanding of target biology and disease pathology by (1) Identifying high-dimensional cellular phenotypes in situ, (2) Establishing cell-cell interaction networks based on expected protein interactions and spatial proximity and (3) Linking features of disease histopathology to changes in cell-cell interaction networks and target expression to cell-cell interaction networks and histopathological features of disease

This project will help in our understanding of cell/protein interactions with histopathological features enabling novel target identification. Moreover, spatially resolving target expression may inform choice of drug modality, while leveraging cell/protein interactions may aid in the identification of target engagement biomarkers/assays.

### Ethics and dissemination

The study will be conducted in accordance with ethical principles outlined in the Declaration of Helsinki (1996), as well as the guidelines for GCP and Good Laboratory Practice. It will also adhere to the UK Department of Health Policy Framework for Health and Social Care (2017) and comply with the Human Tissue Act (2004). Ethical approval for the NAZA study was granted by the West of Scotland Research Ethics Committee (REC). REC reference 22/WS/0099, IRAS project ID 306306. Recruitment began in December 2022 and is currently ongoing at 10 NHS Trust sites across the UK. If protocol amendments are necessary, any changes requiring REC approval will not be implemented until the amendment, along with any revised informed consent forms and participant information sheets (if applicable), has been reviewed and received a favourable opinion from the REC. Minor protocol amendments involving only logistical or administrative changes may be implemented immediately, with subsequent notification to the REC.

The process for obtaining informed consent from participants will adhere to the guidance provided by the REC, as well as GCP and any other applicable regulatory requirements. Prior to participation in the study, both the investigator or their nominee and the participant must sign and date the Consent Form. The participant will receive a copy of the signed forms, while the original will be retained in the Study Master File. Additionally, a second copy will be filed in the participant’s medical notes, with a signed and dated entry confirming that informed consent was obtained. No study-specific interventions will take place until informed consent is obtained. Participation in the study is entirely voluntary, and the investigator or their nominee will clearly communicate to participants that they may withdraw their consent at any time without penalty or loss of access to future medical care or benefits to which they are entitled. Should the Consent Form be amended during the course of the study, the investigator will ensure compliance with all regulatory requirements for the approval of the amended form by the REC.

Participants may be discontinued from the NAZA study at any time at the discretion of the investigator, and they are also free to withdraw their participation voluntarily. Information regarding the withdrawal of informed consent will be collected as outlined in the Data Management Plan. If a participant discontinues their participation, their enrolment number cannot be reassigned to another participant. Specific reasons for discontinuation may include withdrawal of informed consent for the use of biological samples collected as part of the study, incorrect inclusion, or the development of an exclusion criterion, such as non-IBD participants whose cancer screening results indicate a confirmed or suspected finding of cancer. Additionally, if a participant is withdrawn due to a suspected infection classified under WHO risk categories 2, 3 or 4, no biological samples from that patient will be analysed further, and the samples will be destroyed following standard procedures at the study site.

Findings from this study will be submitted for publication in peer-reviewed journals and presentation at national and international scientific conferences.

### Patient and public involvement

The protocol and the participant-facing documentation was prepared by the chief investigator and AstraZeneca while supported by an IBD patient panel hosted by the NIHR Nottingham BRC. Patients provided feedback to ensure the patient pathway was least burdensome while maximising data recovery. Patients reviewed and approved all lay documentation to ensure these were clear and understandable by prospective participants.
