# Supplementary material for: Identification of novel molecular drivers, prognostic and diagnostic biomarkers for Inflammatory Bowel Disease (IBD): protocol for the Nottingham/AstraZeneca prospective IBD observational cohort study
Source: BMJ Open. 2025 Nov 23;15(11):e105790. doi: 10.1136/bmjopen-2025-105790 (PMC12645654; doi:10.1136/bmjopen-2025-105790)
Supplement: Supplementary file 1 [file bmjopen-2025-105790.reviewer_comments.pdf]

PEER REVIEW HISTORY

BMJ Open publishes all reviews undertaken for accepted manuscripts. Reviewers are asked to complete a checklist review form and are provided with free text boxes to elaborate on their assessment. These free text comments are reproduced below.

This paper was submitted to another journal from BMJ but declined for publication following peer review. The authors addressed the reviewers' comments and submitted the revised paper to BMJ Open. The paper was subsequently accepted for publication at BMJ Open.

ARTICLE DETAILS

Title (Provisional)

Identification of novel molecular drivers, prognostic and diagnostic biomarkers for Inflammatory Bowel Disease (IBD): Protocol for the Nottingham/AstraZeneca prospective IBD observational cohort study.

Authors

Serna-Valverde, Ana Lilia; Rodriguez-Suarez, Eva; Marks, Daniel JB; Gehrmann, Ulf; Neisen, Jessica; Clarke, Sarah; Chew, Thean Soon; Cummings, Fraser; De Silva, Shanika; Gordon, John Nicholas; Knight, Paul; Limdi, Jimmy; Patel, Kamal; Crooks, Benjamin; Sebastian, Shaji; Polytarchou, Christos; Hannan, Nicholas R.F; Moran, Gordon

---

VERSION 1 - REVIEW

**Reviewer**	**1**
**Name**	**Reviewer 1**
**Affiliation**	
**Date**	**27-May-2025**
**COI**	

Comments not published pending reviewer's consent.

VERSION 2 - REVIEW

Reviewer **2**

Name **Weidner, Jens**

Affiliation **Technische Universität Dresden, Medizinische Fakultät "Carl Gustac Carus" Health Sciences/ Public Health**

**Date**	**16-Jul-2025**
**COI**	**None**

Thank you for submitting the protocol titled “Identification of Novel Molecular Drivers, Prognostic and Diagnostic Biomarkers for IBD: Protocol for the Prospective Observational IBD Study by the University of Nottingham/AstraZeneca” for review.

This study represents an important and highly relevant contribution to the field of personalized therapy for IBD. Given the substantial unmet need for more effective treatment strategies and the high costs associated with therapy failure, the approach of identifying molecular markers predictive of response to advanced therapies is of great significance. The primary aim to generate comprehensive molecular profiles from various sample types and correlate them with treatment response is highly timely and has the potential to be a key step toward the development of precision medicine in IBD.

The methodology is clearly and comprehensively described. The study design, as a multicenter prospective cohort study, is robust, and the detailed description of sample collection, processing, and storage suggests high data quality. The choice of study timepoints and the extensive phenotyping of participants are also well-justified.

The sample size calculation appears reasonable. Although the exploratory nature of the analysis makes precise sample size estimation challenging, the planned cohort size of 240 participants divided across different treatment arms and the healthy control group seems appropriate for detecting biologically meaningful differences, as outlined in the protocol.

The in-text references need to be revised.

**Reviewer**	**3**
**Name**	**Pritzker, Kenneth**
**Affiliation**	**University of Toronto**
**Date**	**27-Sep-2025**
**COI**	**None**

Some suggestions:

Eligibility criteria:Controls should have same baseline analyses of serum and fecal analytes and be in the exclusion range compared to IBD group.

Duration of relevant symptoms prior to study including before IBD diagnosis, should be recorded.

A caution: For CD patients and for long standing UC patients, mucosal biopsies may not be representative of most active disease, as disease activity may be centered in deeper tissues..

Further in chronic IBD on therapy, biomarkers of repair activity (fibrosis) as well as inflammation markers should be observed.

VERSION 2 - AUTHOR RESPONSE

Reviewer 2: Mr. Jens Weidner, Technische Universität Dresden
Comment 1: The reviewer provided positive feedback on the study's design, methodology, and scientific rationale, noting its relevance and contribution to precision medicine in IBD.
Response: We are grateful for the reviewer's positive assessment of our study's design and objectives.

Comment 2: The in-text references need to be revised.
Response: The in-text references have been reviewed and corrected where necessary. The five referencing errors previously identified have now been resolved.

Reviewer 3: Dr. Kenneth Pritzker, University of Toronto
Comment 1: Eligibility criteria: Controls should have same baseline analyses of serum and fecal analytes and be in the exclusion range compared to IBD group.
Response: We thank the reviewer for this suggestion. While modifying the eligibility criteria at this stage would not be feasible, as recruitment is ongoing and 182 participants have already been enrolled, we agree that ensuring appropriate comparability between groups is important. We will therefore address this consideration during the analysis phase to account for potential differences between the healthy controls and the IBD group.

Comment 2: Duration of relevant symptoms prior to study including before IBD diagnosis, should be recorded.
Response: We appreciate this comment, unfortunately, this information is not consistently available for all participants and was not part of the original data capture plan. Adding this variable retrospectively would risk introducing inconsistencies. Therefore, it is not possible to include this measure at this stage of the study.

Comment 3: A caution- For CD patients and for long standing UC patients, mucosal biopsies may not be representative of most active disease, as disease activity may be centered in deeper tissues. Further in chronic IBD on therapy, biomarkers of repair activity (fibrosis) as well as inflammation markers should be observed.
Response: We thank the reviewer for this valuable caution and fully acknowledge that mucosal biopsies may not fully capture deeper disease activity. This limitation, along with fibrosis-related biomarkers, will be taken into account in the interpretation of findings and addressed in subsequent analyses and reports arising from the study.

Once again, we sincerely thank you and reviewers for the constructive feedback, and hope that the revised version addresses all comments satisfactorily.
